# When I Move, You Move: Physical Activity and Cognitive Functioning Among Older Dyads in Europe

**DOI:** 10.1093/geroni/igaf122.2570

**Published:** 2025-12-31

**Authors:** Laura Himmelmann, Jeffrey Stokes, Tim Stuckenschneider, Tania Zieschang, Kathrin Boerner

**Affiliations:** The Carl von Ossietzky University of Oldenburg’s School of Medicine and Health Sciences, Oldenburg, Niedersachsen, Germany; University of Massachusetts Boston, Boston, Massachusetts, United States; The Carl von Ossietzky University of Oldenburg’s School of Medicine and Health Sciences, Oldenburg, Niedersachsen, Germany; The Carl von Ossietzky University of Oldenburg’s School of Medicine and Health Sciences, Oldenburg, Niedersachsen, Germany; Carl von Ossietzky University of Oldenburg, Oldenburg, Niedersachsen, Germany

## Abstract

Alzheimer’s disease and related dementias (ADRD) impair cognitive function and well-being in older adults. While physical activity helps maintain cognitive health, less is known about the role of a partner’s activity regarding cognitive outcomes. Theories of social contagion and social control suggest that having an active partner may promote greater engagement in physical activity, benefiting cognitive function. However, research using sensor-based measures to investigate physical activity in dyads remains limited. This study examines how physical activity within dyadic relationships predicts cognitive function. Specifically, we assessed how individuals’ and their spouses’ physical activity influenced cognitive outcomes over two years using data from the Survey of Health, Ageing, and Retirement in Europe (SHARE). A two-wave dyadic analysis was conducted with n = 60 older adults from 33 opposite-sex couples (mean age 67.7 ± 8.7 years) in European countries. Physical activity was measured by a three-axial accelerometer, and cognitive function was assessed through verbal fluency at baseline and follow-up. Multilevel LDV modeling with mixed effects models was performed in Stata Version 18/SE. Verbal fluency remained stable over time (β = 0.62, p < .001). Higher step counts predicted better cognitive function at follow-up (β = 0.30, p < .001). Additionally, spouses’ physical activity positively correlated with individuals’ cognitive function at follow-up (β = 0.25, p < .01). Our findings underscore the role of dyadic interactions in healthy aging. Future research should explore underlying mechanisms and assess dyadic interventions to support cognitive well-being in older adults.

